# Determination of Ascorbic Acid Content of Some Fruit Juices and Wine by Voltammetry Performed at Pt and Carbon Paste Electrodes

**DOI:** 10.3390/molecules16021349

**Published:** 2011-02-01

**Authors:** Aurelia Magdalena Pisoschi, Aneta Pop, Gheorghe Petre Negulescu, Aurel Pisoschi

**Affiliations:** 1 Chemistry and Biochemistry Department, University of Agronomic Sciences and Veterinary Medicine of Bucharest, 59, Marasti Street, 011464, Bucharest, Romania; 2 Faculty of Natural Sciences, Vasile Goldis Western University, 94, Revolutiei Avenue, Arad, Romania

**Keywords:** differential pulse voltammetry, cyclic voltammetry, ascorbic acid, juices, Pt electrode, carbon paste electrode

## Abstract

A method was developed for assessing ascorbic acid concentration in fruit juices and wine by differential pulse voltammetry. The oxidation peak for ascorbic acid occurs at about 530 mV (versus SCE) on a Pt strip working electrode and at about 470 mV on a carbon paste working electrode. The influence of the operational parameters like the pulse amplitude and the pulse period on the analytical signal was investigated. The obtained calibration graph shows a linear dependence between the peak height and ascorbic acid concentration within the range 0.31-20 mM with a Pt working electrode, and within the range 0.07-20 mM with a carbon paste working electrode. The equation of the calibration graph was y = 21.839x + 35.726, r^2 ^= 0.9940, when a Pt strip electrode was used (where y represents the value of the current intensity measured for the peak height, expressed as µA and x the analyte concentration, as mM). R.S.D. = 2.09%, n = 10, C_ascorbic acid_ = 2.5 mM. The equation of the calibration graph was y = 3.4429x + 5.7334, r^2^ = 0.9971, when a carbon paste electrode was used (where y represents the value of intensity measured for the peak height, expressed as µA and x the analyte concentration, as mM). R.S.D. = 2.35%, n = 10, C_ascorbic acid_ = 2.5 mM. The developed method was applied to ascorbic acid assessment in fruit juices and wine. The ascorbic acid content determined ranged between 6.83 mg/100 mL juice for soft drinks (Fanta Madness) and 54.74 mg/100 mL for citrus (lemon) juices obtained by squeezing fruit. Different ascorbic acid concentrations (from standard solutions) were added to the analysed samples, the degree of recovery being comprised between 94.74 and 104.97%. The results of ascorbic acid assessment by differential pulse voltammetry were compared with those obtained by cyclic voltammetry. The results obtained by the two methods were in good agreement.

## 1. Introduction

Vitamin C is a water-soluble, antioxidant vitamin. It is important in forming collagen, a protein that gives structure to bones, cartilages, muscles, and blood vessels. Vitamin C also aids in the absorption of iron, and helps maintain capillaries, bones, and teeth. It is the most common electroactive biological compound and one of the most ubiquitous vitamins ever discovered. Rich sources include blackcurrant, citrus fruit, leafy vegetables, tomatoes, green and red peppers. Ascorbic acid is known for its reductive properties. Hence, it is used on a large scale as antioxidant in food and drinks [1]. Due to its content variation caused by the thermal lability, vitamin C represents an important quality indicator that contributes to the antioxidant properties of food [2,3]. 

Traditional methods for ascorbic acid assessment involve titration with an oxidant solution: dichlorophenol indophenol (DCPIP) [4], potassium iodate [5] or bromate [6]. Chromatographic methods, particularly HPLC with electrochemical detection [7], has turned out to be a selective and sensitive method for ascorbic acid assessment in foodstuffs and biological fluids. Fluorimetric methods [8,9] and UV-VIS absorbance-based determinations [10] were also used for ascorbic acid estimation.

The use of a carbon nanotube/ferritin film in the construction of an amperometric biosensor allows the determination of ascorbic acid with a sensitivity of 767 µA/mg (for a 1 mmole L^-1^ vitamin C solution). The ferritin protein bound to single-wall carbon nanotubes enhances the oxidation reaction of ascorbic acid over 11-fold [11]. 

A biosensor based on laccase immobilized on an electrode modified with a self-assembled monolayer, allowed ascorbic acid determination with a detection limit of 1.4 nM [12]. Oxygen permeable hydrophobic ascorbate oxidase micelle membranes were coated on both aminated glassy carbon electrode and gold electrode for the amperometric detection of ascorbic acid, based on the consumption of oxygen. These biosensors have good sensitivities with short response time (within 1 min.) [13]. An amperometric biosensor was developed for ascorbic acid determination in fruit juices, by immobilizing ascorbate oxidase on a nylon membrane, which was fixed on a Clark-type transducer [14].

A recently developed voltammetric methods allow rapid, simple, selective and sensitive determination of low molecular weight antioxidants and vitamins (e.g., ascorbic acid) and drugs, without the necessity of time consuming separation [1,15]. 

Square-wave voltammetry was used to determine ascorbic acid, based on its oxidation at a zeolite modified carbon paste electrode [16]. Cyclic and differential pulse voltammetry were used for electrocatalytical ascorbic acid determination, at a carbon paste electrode, modified with 2,7-bis (ferrocenylethynyl)fluoren-9-one [17]. Cyclic voltammetry at a bare Pt electrode was applied to ascorbic acid content estimation in citrus juices and soft drinks [18].

The electrochemical oxidation and selective determination of ascorbic acid in pharmaceutical dosage forms and in some *Rosa* species was investigated by cyclic, differential pulse and square-wave voltammetry [1]. The linear response was obtained in the range 3.52-176.1 μg mL^-1^, with a detection limit of 0.88 μg mL^-1^ for DPV and 0.52 μg mL^-1^ for SWV. 

Cyclic voltammetry studies performed on Pt electrodes proved that the growth of Pt surface oxides and the anodic response of a variety of interferrents (glucose, cystine, oxalate) was greatly surpressed by the use of fluorosurfactant-modified Pt electrodes [19]. Ascorbic acid was determined in the presence of SO_2_ and acetaldehyde by pulsed voltammetry at interdigitated Pt microelectrodes [20].

Ascorbic acid, uric acid and dopamine were simultaneously determined by differential pulse voltammetry, performed on a glassy carbon electrode modified with a film of poly (3-(5-chloro-2-hydroxyphenylazo)-4,5-dihydroxynaphtalene-2,7-disulphonic acid [21]. Differential pulse voltammetry was used for the assessment of ascorbic acid, at poly (3,4-ethylendioxythiophene)- modified electrodes [22]. 

Simultaneous determination of vitamins C, B_6_ and PP in pharmaceuticals formulations was performed using differential pulse voltammetry at a glassy carbon electrode [23]. Differential pulse voltammetry at a glassy carbon electrode was also employed for the analytical characterization and measuring of predominant flavonoid and phenolic acids [24].

Dopamine was determined in the presence of ascorbic and uric acids by differential pulse voltammetry at a bare glassy carbon electrode [25]. Simultaneous determination of vitamin C and uric acid was also possible with a ferrocenium-thioglycollate modified electrode. Under the optimal conditions and within the linear range of 1 × 10^-6^ M to 5 × 10^-4^ M, the achieved detection limits for ascorbic acid and uric acid were 2 × 10^-7^ M and 1 × 10^-7^ M, respectively [26]. 

A differential pulse voltammetric method at a glassy carbon working electrode was also developed for the determination of Silymarin/vitamin E acetate mixture in pharmaceuticals [27]. The linear analytical response was obtained in the range 0.1-4.0 mg L^-1^, with a detection limit of 0.03 mg L^-1^ for Silymarin, and 0.05-4.0 mg L^-1 ^with a detection limit of 0.01 mg L^-1^ for vitamin C acetate [27]. A multi-walled carbon nanotubes-tetradecyltrimethylammonium bromide film coated graphite electrode was used to study the electrooxidation of ascorbic acid in differential pulse, cyclic and square-wave voltammetry [28]. A linear voltammetric response for vitamin C was obtained for the concentration range 5 × 10^-7^-1.7 × 10^-4^ M, with a detection limit for ascorbic acid of 1.1 × 10^-7^ M, using DPV [28]. 

The electrocatalytic oxidation of ascorbic acid (cyclic and differential pulse voltammetry) was investigated with a carbon nanotube paste electrode modified with 2,2’-[1,2-ethanediylbis (nitriloethylidine)]-bis-hydroquinone [29]. Using DPV, the calibration curves for ascorbic acid and uric acid were obtained over the ranges 0.1-800 μM and 20-700 μM, respectively [29]. Differential pulse voltammetry with a poly(sulfonazo III) modified glassy carbon electrode enables the highly selective determination of ascorbic acid, dopamine and uric acid [30].

Cyclic voltammetry and differential pulse voltammetry at a binuclear copper complex modified glassy carbon electrode were also applied to determine ascorbic acid and dopamine [31]. Linear analytical curves were obtained in the ranges 2.0-120.0 µM for dopamine and 5.0-160.0 µM for ascorbic acid, using DPV. The detection limits were 1.4 × 10^-6^ M for dopamine and 2.8 × 10^-6 ^M for ascorbic acid [31]. The modified electrode was used for ascorbic acid and dopamine determination in medicine and foodstuffs [31].

Ascorbic acid and dopamine were determined simultaneously by differential pulse voltammetry performed at a boron-dopped diamond film electrode [32] or on the surface of electrodes modified with self assembled gold nanoparticles film [33]. The linear analytical curves were obtained in the ranges 0.3-1.4 mM for ascorbic acid and 0.2-1.2 mM for dopamine. The detection limit (3σ) was 9.0 × 10^-5 ^M for both dopamine and ascorbic acid [33]. Differential pulse voltammetry at a glassy carbon electrode was applied to quantitative determination of ascorbic acid in tablet dosage form and in some fruit juices [34]. 

The simultaneous assessment of ascorbic acid and acetaminophen was investigated by differential pulse voltammetry and cyclic voltammetry, performed on a boron-doped diamond electrode, using sodium sulphate as supporting electrolyte [35]. Fouling of the electrode was not reported. Relative standard deviations of 2-3%, high sensitivity values and low detection limits (10^-6^M order of magnitude) were obtained [35]. 

This study aims at investigating the ascorbic acid determination by differential pulse voltammetry at Pt strip and carbon paste working electrodes. The developed method was applied to ascorbic acid content assessment in juices and wine. The results were compared to those obtained by cyclic voltammetry, performed at both Pt and carbon paste electrodes.

## 2. Results and Discussions

### 2.1. Voltammetric studies performed at a Pt working electrode

**Figure 1:**
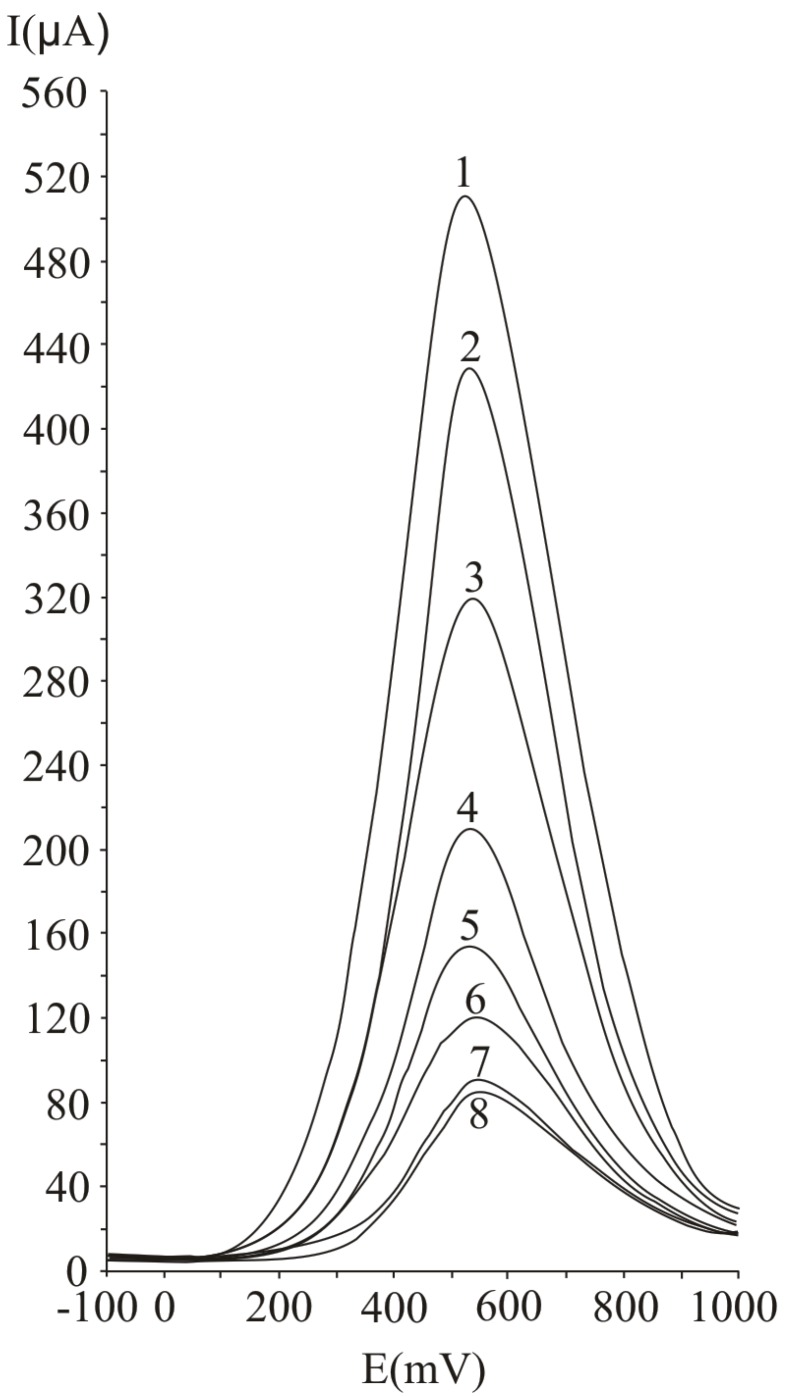
Differential pulse voltammograms obtained with a Pt working electrode for different ascorbic acid concentrations, expressed as mM: 20 (1), 15 (2), 10 (3), 5 (4), 2.5 (5), 1.25 (6), 0.625 (7) and 0.31 (8); experimental conditions: pulse amplitude 75 mV, pulse period 125 ms, potential scan rate 50 mV/s.

**Figure 2:**
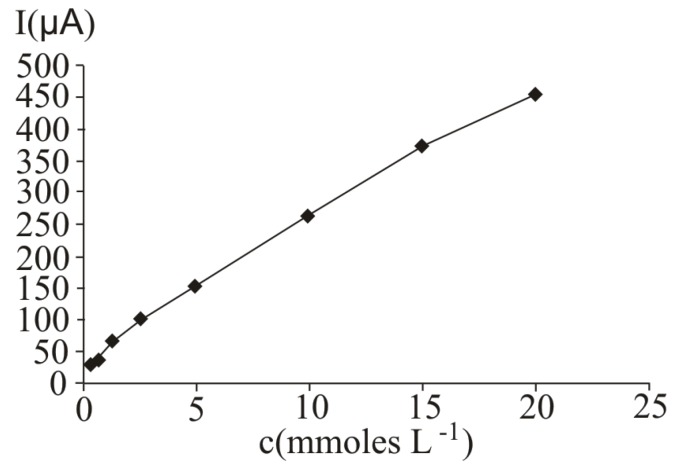
Calibration graph obtained at ascorbic acid determination by differential pulse voltammetry at a Pt working electrode; for experimental conditions see Figure 1.

In Figure 1, several differential pulse voltammograms, obtained obtained at a Pt working electrode, for different ascorbic acid concentrations, are presented. The peak corresponding to ascorbic acid oxidation appeared at 530 mV (versus SCE). The calibration graph (Figure 2) shows a linear range obtained between 0.31 and 20 mM ascorbic acid, (y = 21.839x + 35.726, r^2^ = 0.9940, where y represents the value of the current intensity, from which the background value was substracted and x the analyte concentration). The value calculated for the relative standard deviation R.S.D. was 2.09%, (c = 2.5 mM ascorbic acid; n = 10). The values obtained for the limit of detection and the limit of quantification were 0.087 mM and 0.29 mM respectively.

The detection limit was calculated as LOD = 3 s/m, where s represents the square mean error calculated for 10 determinations of the blank and m represents the slope of the calibration graph. The limit of quantification was calculated as LOD = 10 s/m, where s and m were given above.

The influences of the pulse amplitude and pulse period on the analytical peak height were also investigated. The measurements were performed at 10 mM ascorbic acid concentration, at a 50 mV s^-1^ potential scan rate. 

**Figure 3:**
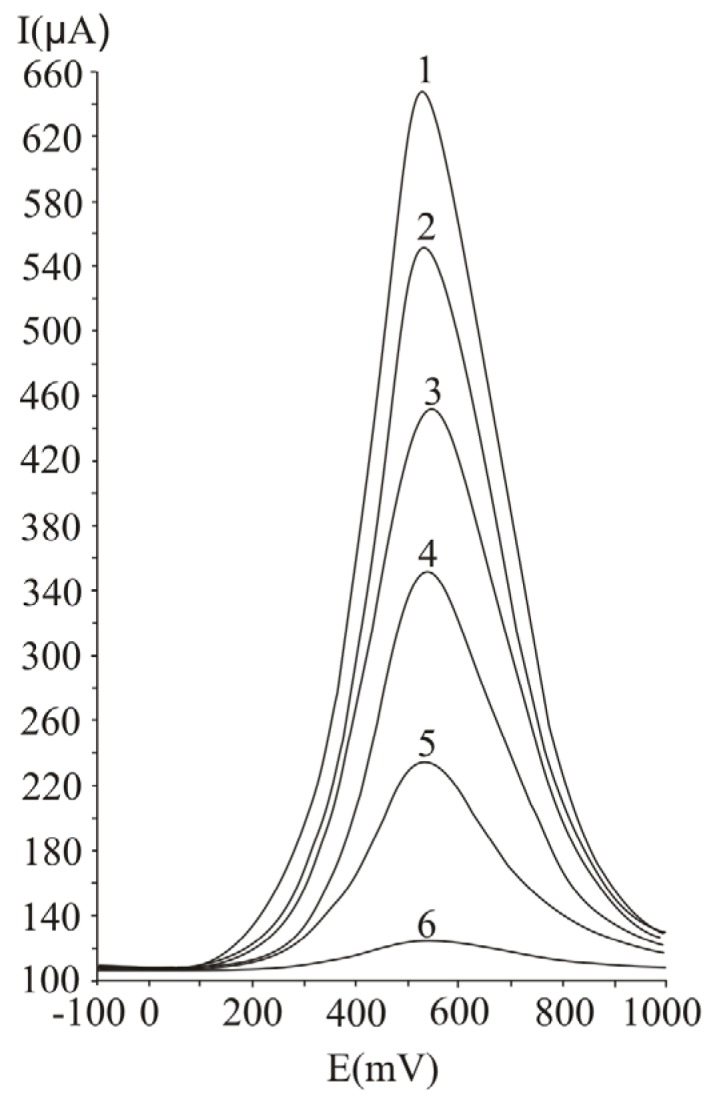
Influence of the pulse amplitude on the analytical response at ascorbic acid determination by differential pulse voltammetry, at a Pt working electrode; 25 mV (1), 50 mV (2), 75 mV (3), 100 mV (4), 125 mV (5) and 150 mV (6); experimental conditions: pulse amplitude 125 ms, potential scan rate 50 mV/s.

For the investigation of the influence of the pulse amplitude on the analytical signal (Figure 3), we varied this parameter between 25 and 150 mV, at 100 ms pulse period and 50 mV/s potential scan rate. By analysing the results presented in Figure 3, it can be noticed that the value of the measured current intensity increases with the applied pulse amplitude. An optimum value of 75 mV was chosen for further studies and for real sample analysis. Greater values of the pulse amplitude were not employed, in order to avoid the decrease of resolution. 

**Figure 4:**
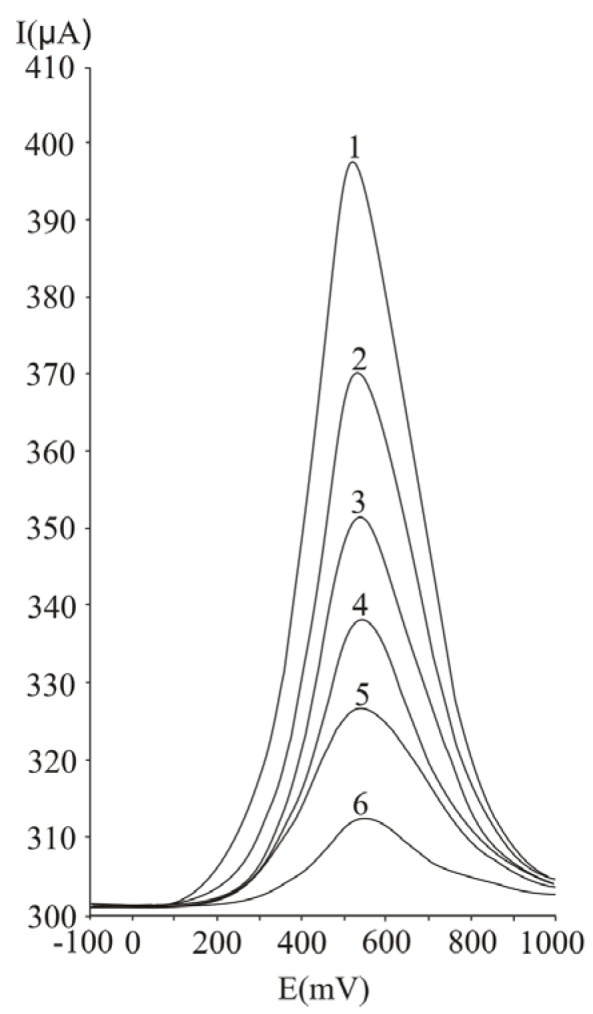
Influence of the pulse period on the analytical signal at ascorbic acid determination by differential pulse voltammetry, at a Pt working electrode; 50 ms (1), 75 ms (2), 100 ms (3), 125 ms (4), 150 ms (5); experimental conditions: pulse amplitude 75 mV, potential scan rate 50 mV/s.

For the investigation of the influence of the pulse period on the analytical signal (Figure 4) we varied this parameter between 25 and 150 mV, at 75 mV pulse amplitude and 50 mV s^-1^ potential scan rate. The peak height increases with the decrease of the pulse amplitude. The value chosen for further studies was 125 ms. Smaller values of the pulse amplitude were not applied, in order to diminish the influence of noise on the analytical signal, noticeable, namely, at values of the pulse period below 100 ms.

**Figure 5:**
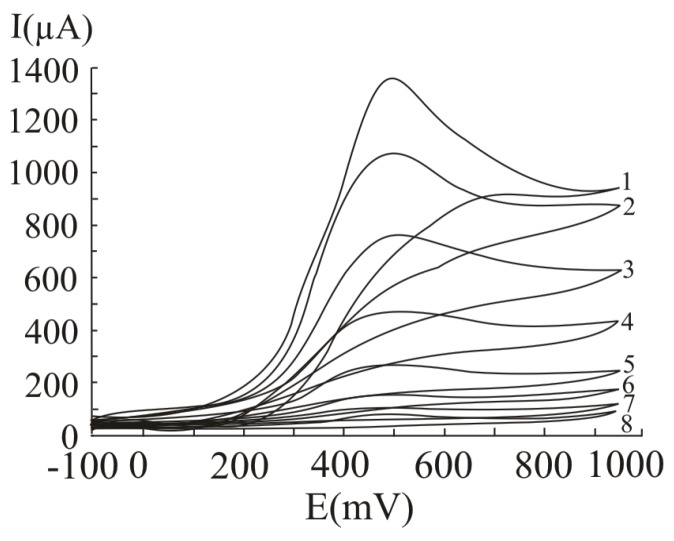
Cyclic voltammograms obtained with a Pt working electrode for different ascorbic acid concentrations, expressed as mM: 20 (1), 15 (2), 10 (3), 5 (4), 2.5 (5), 1.25 (6), 0.625 (7) and 0.31 (8); potential scan rate 50 mV/s.

**Figure 6:**
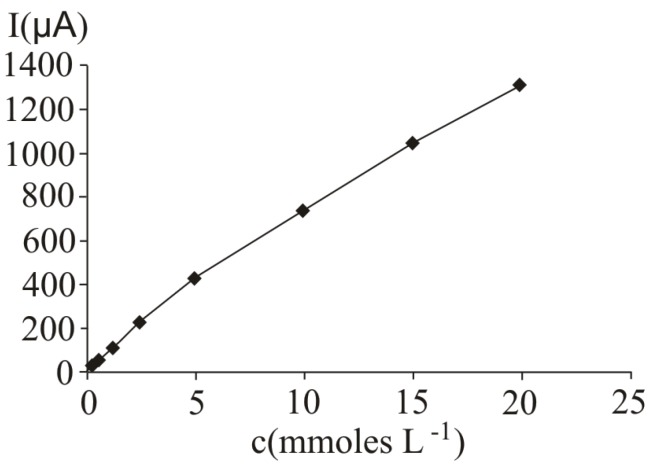
Calibration graph obtained at ascorbic acid determination by cyclic voltammetry at a Pt working electrode; for experimental conditions see Figure 5.

In Figure 5, several cyclic voltammograms, obtained for different ascorbic acid concentrations, are presented. The peak corresponding to ascorbic acid oxidation appeared at 490 mV (versus SCE). The calibration graph obtained by cyclic voltammetry (Figure 6) shows a linear range obtained between 0.31 and 20 mM ascorbic acid (y = 65.42x + 40.887, r^2^ = 0.9945, where y represents the value of the current intensity, from which the background value was substracted and x the analyte concentration). The value calculated for R.S.D. was 1.64%, (c = 2.5 mM ascorbic acid; n = 10). The limit of detection and the limit of quantification were 0.075 mM and 0.25 mM respectively.

### 2.2. Voltammetric studies performed at a carbon paste working electrode

**Figure 7:**
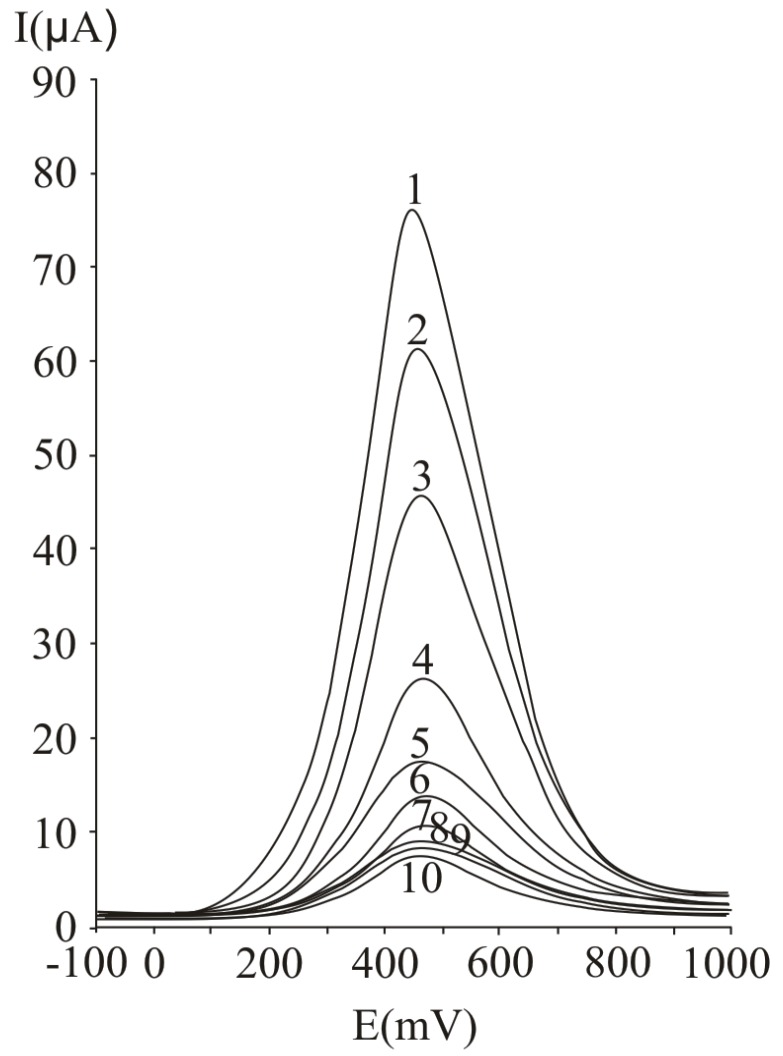
Differential pulse voltammograms obtained with a carbon paste working electrode for different ascorbic acid concentrations, expressed as mM: 20 (1), 15 (2), 10 (3), 5 (4), 2.5 (5), 1.25 (6), 0.625 (7), 0.31 (8) 0.15 (9) and 0.07 (10); experimental conditions: pulse amplitude 75 mV, pulse period 125 ms, potential scan rate 50 mV/s.

**Figure 8:**
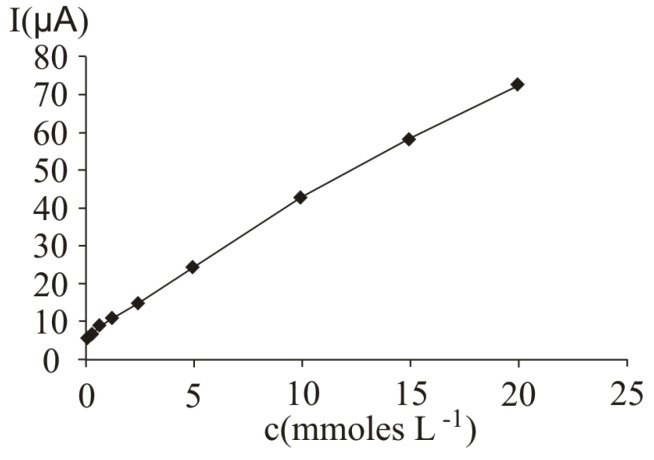
Calibration graph obtained obtained at ascorbic acid determination by differential pulse voltammetry at a carbon paste working electrode; for experimental conditions see Figure 7.

In Figure 7, several differential pulse voltammograms, obtained with a carbon paste electrode for different ascorbic acid concentrations, are presented. The peak corresponding to ascorbic acid oxidation appeared at 470 mV (versus SCE). The calibration graph (Figure 8) shows a linear range obtained between 0.07 and 20 mM ascorbic acid (y = 3.4429x + 5.7334, r^2^ = 0.9971, where y represents the value of the current intensity, from which the background value was substracted and x represents the analyte concentration). The value calculated for R.S.D. was 2.35%, (c = 2.5 mM ascorbic acid; n = 10). The limit of detection and the limit of quantification were 0.02 mM and 0.068 mM respectively.

The results obtained are close to the ones previously reported in literature: the detection limit at ascorbic acid determination by DPV at a modified glassy carbon electrode was 2.1 × 10^-5^ M [36].

The optimum value of 75 mV was used for the pulse amplitude and 125 ms was the value chosen for the pulse period, for the reasons explained at ascorbic acid determination at a Pt working electrode. 

**Figure 9:**
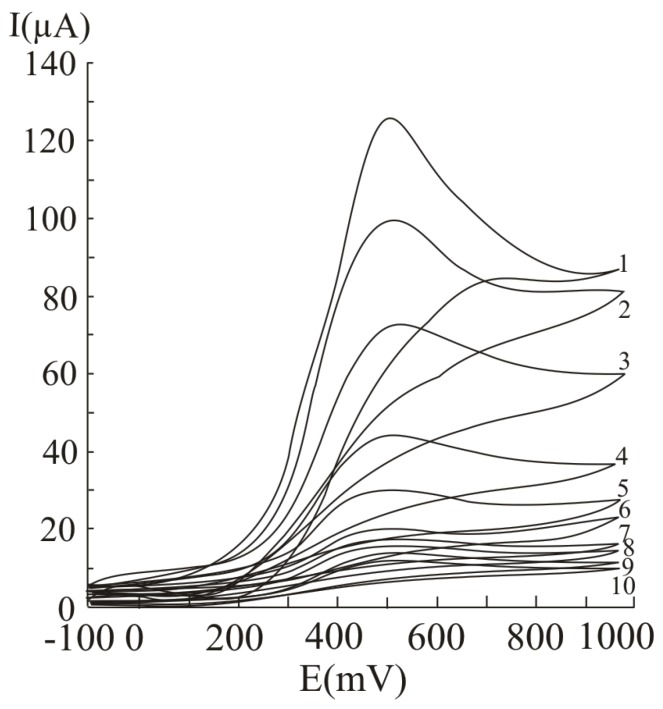
Cyclic voltammograms obtained with a carbon paste working electrode for different ascorbic acid concentrations, expressed as mM: 20 (1), 15 (2), 10 (3), 5 (4), 2.5 (5), 1.25 (6), 0.625 (7), 0.31 (8), 0.15 (9) and 0.07 (10); potential scan rate 50 mV/s.

**Figure 10:**
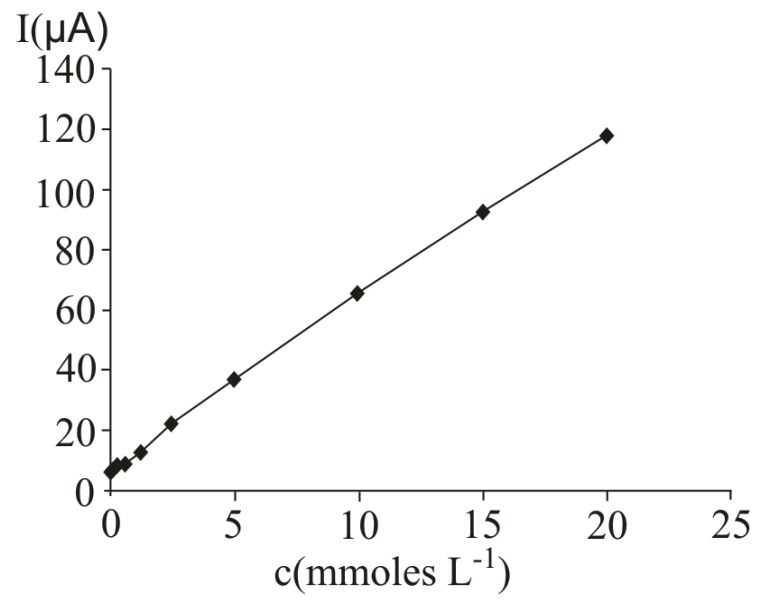
Calibration graph obtained obtained at ascorbic acid determination by cyclic voltammetry at a carbon paste working electrode; for experimental conditions see Figure 9.

In Figure 9, several cyclic voltammograms, obtained for different ascorbic acid concentrations, are presented. The peak corresponding to ascorbic acid oxidation appeared at 510 mV (versus SCE). The calibration graph obtained by cyclic voltammetry (Figure 10) presents a linear range between 0.07 and 20 mM ascorbic acid (y = 5.674x + 6.5619, r^2^ = 0.9988, where y represents the value of the current intensity, from which the background value was substracted and x is the analyte concentration). The value calculated for R.S.D. was 2.29% (c = 2.5 mM ascorbic acid; n = 10). The limit of detection and the limit of quantification were 0.062 mM and 0.018 mM respectively.

### 2.3. Interference study

In order to verify the existence of matrix effects on ascorbic determination by differential pulse voltammetry, the influence of some interferents commonly present in fruit juices, wines and soft drinks, namely citric acid, glucose, tartaric acid and benzoate, was investigated. The influence of the above mentioned compounds on the analytical peak height is presented in Table 1 and Table 2. 

**Table 1:** Study of interferences at ascorbic acid determination by DPV at a Pt electrode, performed on some chemical species commonly found in fruit juices, soft drinks and wines.

Interferent	Interferent/analyte molar ratio	Influence on the analytical peak height
**Glucose**	150	less than 3%
	200	4.8% decrease
**Citric acid**	25	less than 3%
	50	3.9% decrease
	100	5.2% decrease
	150	6.4% decrease
	200	7.9% decrease
**Tartaric acid**	50	less than 3%
	100	5.3% increase
	150	6.1% increase
	200	9.5% increase
**Benzoate anion**	100	less than 3%
	150	4.7 % decrease
	200	6.9 % decrease

**Table 2:** Study of interferences at ascorbic acid determination by DPV at a carbon paste electrode, performed on some chemical species commonly found in fruit juices, soft drinks and wines.

Interferent	Interferent/analyte molar ratio	Influence on the analytical peak height
**Glucose**	200	less than 3%
**Citric acid**	50	less than 3%
	100	3.6 % decrease
	150	4.2 % decrease
	200	5.8 % decrease
**Tartaric acid**	50	less than 3%
	100	3.8 % increase
	150	5.5 % increase
	200	6.7% increase
**Benzoate anion**	100	less than 3%
	150	5.7 % decrease
	200	8.1% decrease

As it can be seen from Table 1 and Table 2, citric acid, tartaric acid and benzoate anion have no significant influence on the analytical signal, up to an interferent/analyte molar ratio of 50/1. Glucose has no influence on the analytical response, in concentrations up to 150 times greater than that of vitamin C.

Therefore, at concentrations commonly found in juices, soft drinks and wines, these organic compounds do not give significant interferences, at ascorbic acid assessment by the investigated voltammetric method. 

### 2.4. Real sample analysis

Natural juices (commercial and freshly obtained from fruit) containing pulp were centrifugated before analysis. Soft drinks did not necessitate centrifugation. Solid potassium chloride was added as supporting electrolyte to the undiluted clear sample, in order to obtain a concentration of 0.10 M KCl. The working procedure employed for standard ascorbic acid solutions was also applied to juice and wine analysis by differential pulse voltammetry and cyclic voltammetry. 

For the determination of the degree of recovery of vitamin C added to the analysed juice and wine samples, 1 mL (17.6 mg), from a concentrated (0.10 M) ascorbic acid solution containing 0.10 M KCl, was added to 50 mL sample. The obtained analytical signal was corrected by taking into account the sample dilution originating from the addition of standard ascorbic acid solution. For each addition, the degree of recovery was calculated:

*Recovery% = (Q_DET_-Q_P_) × 100/ Q_ADD_*

where Q_DET _represents mg ascorbic acid determined in 100 mL juice, Q_P_ represents mg ascorbic acid previously present in 100 mL juice and Q_ADD, _mg ascorbic acid added in 100 mL juice. The obtained results are presented in Table 3 and Table 4.

**Table 3:** Results obtained at ascorbic acid determination in natural juices, soft drinks and wines by differential pulse voltammetry and cyclic voltammetry performed at a Pt strip working electrode.

Analysed product (producer)	Ascorbic acid concentration-cyclic voltammetry (mg/100 mL juice)	Ascorbic acid concentration -cyclic voltammetry- after addition (35.2 mg AA added to 100 mL juice)	Degree of recovery (%)	Ascorbic acid concentration- differential pulse voltammetry (mg/100 mL juice)	Ascorbic acid concentration -differential pulse voltammetry- after addition (35.2 mg AA added to 100 mL juice)	Degree of recovery (%)
Orange juice (fruit pressing)	39.25	72.25	97.86	41.24	76.06	103.24
Grapefruit juice (fruit pressing)	35.23	70.43	103.99	39.82	74.54	102.87
Lemon juice (fruit pressing)	50.82	85.96	104.72	52.15	85.19	98.72
Fanta Madness (Coca Cola HBC Romania)	7.26	42.80	103.42	6.83	42.27	103.10
Prigat active grapefruit (Quadrant Amroq Beverages)	12.46	47.18	101.33	11.79	44.51	95.48
Prigat active sour cherry (Quadrant Amroq Beverages)	11.91	48.15	105.68	12.38	46.99	101.01
Prigat kiwi (Quadrant Amroq Beverages)	14.14	47.16	96.49	13.93	48.76	101.73
Frutia tomato (European Drinks)	18.92	54.52	104.23	19.41	51.72	94.73
Wine (Recas vineyard)	15.86	48.42	95.25	15.66	51.49	104.72

**Table 4:** Results obtained at ascorbic acid determination in natural juices, soft drinks and wines^* ^by differential pulse voltammetry and cyclic voltammetry performed at a carbon paste electrode.

Analysed product	Ascorbic acid concentration -cyclic voltammetry (mg/100 mL juice)	Ascorbic acid concentration -cyclic voltammetry- after addition (35.2 mg AA added to 100 mL juice)	Degree of recovery (%)	Ascorbic acid concentration -differential pulse voltammetry (mg/100 mL juice)	Ascorbic acid concentration- differential pulse voltammetry- after addition (35.2 mg AA added to 100 mL juice)	Degree of recovery (%)
Orange juice	39.40	72.52	98.23	40.68	75.64	103.62
Grapefruit juice	37.71	72.22	102.14	39.05	74.50	104.96
Lemon juice	52.94	87.07	101.91	54.74	87.15	97.04
Fanta madness	7.19	40.77	97.71	7.08	42.58	103.27
Prigat grapefruit	12.81	48.48	104.11	12.27	45.30	96.41
Prigat sour cherry	12.99	45.47	94.87	12.16	44.75	95.13
Prigat kiwi	15.48	50.73	103.04	15.17	49.90	101.51
Frutia tomato	18.63	52.45	99.07	19.24	54.27	102.61
Wine (Recas vineyard)	15.07	48.71	98.35	16.22	49.63	97.74

* Producers are the same as in Table 3.

The ascorbic acid content (Table 3 and Table 4) ranged between 6.83 mg/100 mL sample for soft drinks (Fanta Madness) and 54.74 mg/100 mL sample for citrus (lemon) juices obtained by fruit squeezing. The results obtained by the two methods (differential pulse and cyclic voltammetry) are in good agreement. They are also in good agreement with the data reported in literature regarding the ascorbic acid content of fruit juices: the vitamin C content of tomatoes determined by differential pulse voltammetry at a modified carbon paste electrode was 18.68 mg/100 g sample [31]. The ascorbic acid amount in oranges (*Citrus aurantium*) varied from 30 to 56 mg/100 g of fresh weight [37]. The ascorbic acid content in lemon (*Citrus limon*), estimated by cyclic voltammetry and titrimetry with N-bromosuccinimide was 49.0 and 49.53 mg/100 mL respectively [38]. Melo *et al*. [39], using the 2,6-dichlorophenolindophenol titrimetric method, reported an ascorbic acid content of 37.34 mg/100 g for orange.

### 2.5. Application of the standard addition method at ascorbic acid determination by differential pulse voltammetry performed at a carbon paste electrode

**Figure 11:**
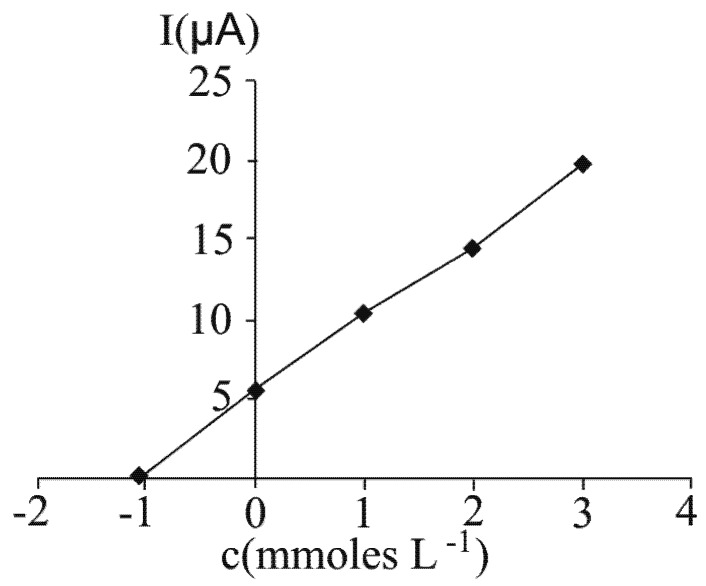
Application of the standard addition method at ascorbic acid determination in fresh grapefruit juice by DPV at carbon paste electrode.

In order to verify the accuracy of the developed method for ascorbic acid determination in fruit juices, the standard addition method was applied to fresh grapefruit juice analysis. The following procedure was employed: to four 100 mL volumetric flasks, 50 mL sample were added. Then, known amounts from the standard 100 mM ascorbic acid solution were added in each flask, as follows: 1) 0 mL; 2) 1 mL; 3) 2 mL and 4) 3 mL. Solid KCl was added in each volumetric flask, as to reach a 0.10 M final electrolyte concentration. Distilled water was added to the final 100 mL volume, followed by homogenisation. The ascorbic acid content of each flask was determined and the obtained results are presented in Figure 11. The measured concentration in the diluted fresh grapefruit juice was 1.06 mM (18.65 mg/100 mL) ascorbic acid. Taking into account the dilution degree (1/1), this corresponds to a concentration of 37.30 mM ascorbic acid in the undiluted grapefruit juice. The obtained result is in accordance with the one presented in Table 4. This indicates the absence of matrix effects at ascorbic acid determination, by the investigated voltammetric method.

## 3. Experimental

### 3.1. Reagents and instrumentation

A KSP potentiostat-galvanostat, laboratory made by Professor Slawomir Kalinowski, University Warmia and Mazury (Olsztyn), as well as the respective softwares, Differential Pulse Voltammetry and Cyclic Voltammetry. A Pt strip electrode (Radelkis 30 mm^2^ surface) and then a carbon paste electrode were used as working electrodes. The reference electrode was a saturated calomel electrode (SCE). The counter electrode was a Pt strip (Radelkis 30 mm^2^ surface). 

**Figure 12:**
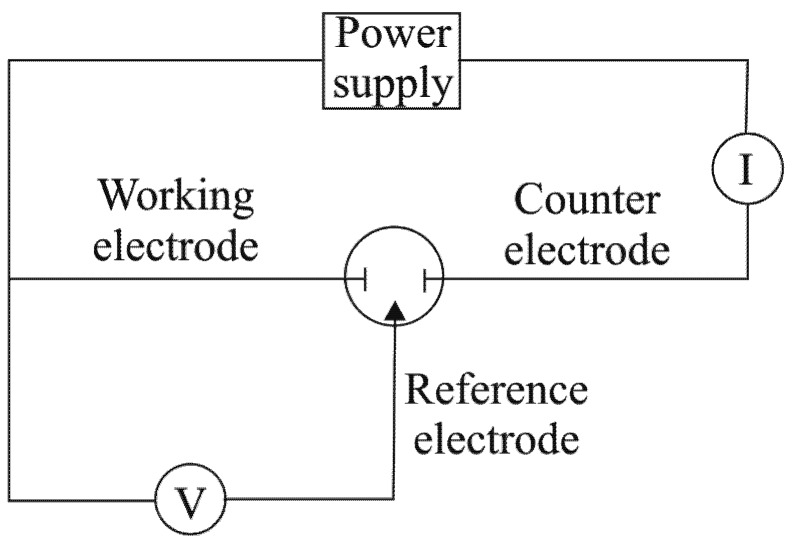
Schematic representation of the experimental setup.

Figure 12 provides a schematic representation of the experimental setup: the potentiostat enables control of the potential of the working electrode, with respect to the reference electrode, as well as measurement of the current that flows between the working electrode and counter electrode. 

A stock solution of ascorbic acid (0.10 M) was prepared daily by dissolving vitamin C (Merck, ACS ISO, biochemical grade) in a 0.10 M KCl solution (Chimopar, Bucharest, Romania), used as supporting electrolyte. Standard solutions of ascorbic acid with concentrations ranging between 0.3 and 20 mM were obtained by diluting the stock solution with the respective volumes of 0.10 M KCl (electrolyte) solution. For the interference studies, glucose and citric acid were purchased from Chimopar (Bucharest, Romania), tartaric acid from Merck (Darmstadt, Germany) and sodium benzoate from Sigma Aldrich (Steinheim, Germany). 

### 3.2. Working procedure

For voltammetric measurements, a three-electrodes cell was used, equipped with working, counter and a reference electrodes. The volume of the analysed sample was 50 mL and all measurements were performed at 295.5 K, using a 0.10 M KCl solution as supporting electrolyte. Before each determination, the Pt working electrode was cleaned mechanically, by applying a -1.5 V potential pulse for 3 seconds. For the differential pulse voltammetry measurements the potential was scaned within the range -100 to 1,000 mV. For the investigation of the influence of the operational parameters on the analytical signal, the pulse amplitude varied between 25 and 150 mV and the pulse period ranged between 50 and 150 ms. For the cyclic voltammetry measurements, the potential was scaned within the range -100 to 1,000 mV, with a 50 mV/s scan rate. The value of the backround current, obtained for the KCl 0.10 M solution was substracted from the current corresponding to the analysed solution/sample, for both voltammetric methods. 

## 4. Conclusions

The developed voltammetric methods (differential pulse voltammetry and cyclic voltammetry) for ascorbic acid determination are characterized by sensitivity, rapidity and reproducibility. The degree of accuracy of the investigated voltammetric methods is confirmed by the values obtained for the degree of recovery, which ranged between 94.74 and 104.97%.

The limit of detection (LOD) and the limit of quantification (LOQ) obtained by differential pulse voltammetry were 0.087 mM and 0.29 mM respectively, when a Pt electrode was used. The limit of detection (LOD) and the limit of quantification (LOQ) obtained by cyclic voltammetry were 0.075 mM and 0.25 mM, respectively. 

Lower LOD and LOQ values were obtained when a carbon paste electrode was employed as working electrode: the limit of detection and the limit of quantification (LOQ) obtained by differential pulse voltammetry were 0.02 mM and 0.068 mM, respectively. The limit of detection and the limit of quantification (LOQ) obtained by cyclic voltammetry were 0.018 mM and 0.062 mM respectively. 

The sensitivity obtained by cyclic voltammetry (given by the slope of the calibration graph) is better than the one obtained by differential pulse voltammetry, with both working electrodes. Differential pulse voltammetry has turned out to be a technique characterized by sensitivity, rapidity, good specificity and reproducibility, and can be applied with good results in food quality control.
